# A Preliminary Cost-Utility Analysis of the Prosthetic Care Innovations: Case of the Keep Walking Implant

**DOI:** 10.33137/cpoj.v4i2.36366

**Published:** 2021-09-21

**Authors:** L Guirao, B Samitier, L Frossard

**Affiliations:** 1 Servicio de Rehabilitaión - Hospital Asepeyo Sant Cugat, Barcelona, Spain.; 2 YourResearchProject Pty Ltd, Brisbane, Australia.; 3 Griffith University, Gold Coast, Australia.; 4 University of the Sunshine Coast, Maroochydore, Australia.; 5 Queensland University of Technology, Brisbane, Australia.

**Keywords:** Artificial Limbs, Bone-Anchored Prosthesis, Cost-Effectiveness, Cost-Utility, Distal Weight Bearing Implant, Osseointegration, Prosthesis

## Abstract

Several obstacles must be overcome before preliminary cost-utility analyses (CUA) of prosthetic care innovations can be routinely performed. The basic framework of preliminary CUAs and hands- on recommendations suggested previously might contribute to wider adoption. However, a practical application for an emerging intervention is needed to showcase the capacity of this proposed preliminary CUA framework. This study presented the outcomes of preliminary CUA of the distal weight bearing Keep Walking Implant (KWI), an emerging prosthetic care innovation that may reduce socket fittings for individuals with transfemoral amputation. The preliminary CUAs compared the provision of prosthetic care without (usual intervention) and with the KWI (new intervention) using a 15-step iterative process focused on feasibility, constructs, analysis, and interpretations of outcomes from an Australia government prosthetic care perspective over a six-year time horizon. Baseline and incremental costs were extracted from schedules of allowable expenses. Baseline utilities were extracted from a study and converted into quality-adjusted life-year (QALY). Incremental utilities were calculated based on sensible gains of QALY from baselines. The provision of the prosthetic care with the KWI could generate an indicative incremental cost-utility ratio (ICUR) of −$36,890 per QALY, which was $76,890 per QALY below willingness-to-pay threshold, provided that the KWI reduces costs by $17,910 while increasing utility by 0.485 QALY compared to usual interventions. This preliminary CUA provided administrators of healthcare organizations in Australia and elsewhere with prerequisite evidence justifying further access to market and clinical introduction of the KWI. Altogether, this work suggests that the basic framework of the preliminary CUA of a prosthetic care innovation proposed previously is feasible and informative when a series of assumptions are carefully considered. This study further confirms that preliminary CUAs prosthetic care interventions might be a relevant alternative to full CUA for other medical treatments.

## INTRODUCTION

### Importance of preliminary CUA of innovation

Developers of new prosthetic care solutions must demonstrate the safety, efficacy, and socio-economic benefits of their innovations.^1–14^ Value for money of a prosthetic care innovation is usually evidenced during a health technology assessment (HTA) and a health economic evaluation (HEE).^14–17^ Providing timely evidence is critical for the clinical promotion of an innovation.

Ijzerman and Steuten (2011) highlighted that an early, preliminary, and full cost-utility analysis (CUA) can be performed at the early, middle, and late stages of clinical acceptance of any medical treatment, respectively.[6] Kannenberg and Seidinger (2019) suggested that these three types of CUAs should be undertaken by manufacturers of prosthetic solutions at the early, middle, and late phases of development.^7^

In Frossard (2021), we noted the consensus around the weaknesses of full CUAs (e.g., lack of timeliness, resource-intensive) and strengths of earlier CUAs (e.g., identify evidence gaps and headroom for improvement, educate full CUA, fast track approval).^13^ Concepts of preliminary CUAs are emerging.[6] However, several obstacles must be overcome before these analyses are routinely performed.^12^ Among others, disparities of methods and high uncertainty make the outcomes of usual preliminary CUAs challenging to interpret, appraise, and share.^6,13,18^

In Frossard (2021), we suggested that these shortcomings could be alleviated by a basic framework of preliminary CUAs, given the already existing standardization of prosthetic care (e.g., list of tasks, timeline of intervention).^18^ Consequently, we put together a basic framework considering fundamentals and applied principles of health economics as well as recent preliminary CUAs of transfemoral and transtibial bone-anchored prostheses.^19,20^ Previously, Frossard (2021) created a 15-step iterative process including hands-on recommendations that focuses on feasibility, constructs, analysis, and interpretations of outcomes. Furthermore, the proposed preliminary CUAs could be facilitated when considering abided constructs, prior schedules of expenses and benchmark of baseline, and incremental utilities.^18^

It was anticipated that this new approach to preliminary CUA could simplify the selection of methods, standardize outcomes, ease comparisons between innovations, and streamline pathways for adoption. However, a practical application for an emerging intervention is required to illustrate and further advance the validation of the proposed basic framework.

### Case of Keep Walking Implant

**Figure 1:**
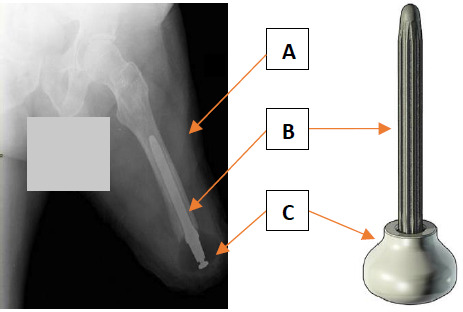
Example of implantation of the distal weight bearing Keep Walking Implant in the transfemoral residuum (A) including and endomedullar femoral stem (B) and rounded spacer (C) that reshaping the distal end of the residuum with a more uniform cone facilitating direct transmission of loading and, altogether, improving safely quality of life and walking ability. ^22–25^

An example of emerging intervention is the distal weight bearing Keep Walking Implant (KWI, TEQUIR S. L., Spain). The KWI involves the surgical insertion of an endomedullar implant including an osseointegrated femoral stem and a rounded spacer into the distal end of residual femur (**Figure 1**). The treatment is indicated for a broad range of case-mix with transfemoral amputation (TFA) due to vascular, trauma, and tumor issues among patients who experience substantial challenges with socket fittings, including non-prosthetic users.^10^ This surgical procedure has been performed gradually on more than 75 cases over the last few years, mainly in Europe and a few other countries.

Preliminary outcomes from ongoing clinical trials suggested that the KWI could potentially ease socket fittings (e.g., reshaping residuum, restore distal weight bearing capability on the femur).^21^ Stronger evidence will be required. Meanwhile, KWI could be reasonably considered as candidate intervention to reduce socket fittings.

### Needs and challenges

The population of individuals suffering from limb loss due to vascular diseases is projected to grow at an unprecedented pace in the next decade worldwide.^26^ There are undeniable market opportunities for solutions, such as the KWI, that can facilitate access to prosthetic fittings for this population. However, evidence of health economics benefits of other interface technologies reducing socket fittings is sparse.^27–29^

Clearly, the KWI is at a stage of development when preliminary evidence of its cost-utility would be most timely and beneficial. Preliminary CUA of the KWI will be particularly appealing for decision-makers involved in advisory committees of governing bodies and healthcare organizations inclined or already familiar with osseointegrated solutions, such as Australia.^8–12,18–20,30–35^

However, any preliminary CUA of the KWI will have to work around the typical sparsity of clinical outcomes collected essentially with a small cohort in a single jurisdiction that is likely to be outside the investigators' own healthcare organization.

### Purposes

The primary purpose of this study was to put the basic framework of preliminary CUA suggested previously to the test with an emerging prosthetic care innovation that could reduce socket fittings for TFAs.^13,18^ The secondary purposes were to:

- Compare ICURs for the provision of transfemoral prostheses fitted to a residuum without (usual treatment) and with the KWI (new treatment) over a mid-term time horizon from an Australian government prosthetic care perspective.
- Establish if the outcomes of this preliminary CUA could be deemed favorable enough to promote further clinical introduction of the KWI in Australia and elsewhere.
- Produce basic information needed to facilitate subsequent primary and modeling CUAs of the KWI (e.g., within-trial horizon studies).

The specific objectives were to:

1) Determine the feasibility of this preliminary CUA, including the evaluation of early evidence of safety and efficacy of the KWI.
2) Outline constructs of this preliminary CUA, including the educated choices made to determine the perspective, time horizon, and various scenarios (e.g., worse-case, best-case, and base-case).
3) Conduct analysis, including ICURs based on estimation of baseline and incremental costs (e.g., schedules of allowable expenses), and utilities (e.g., calculation of retrospective health-related quality of life data, assumption of sensible gains).
4) Interpret outcomes considering the limitations and comparisons with willingness-to-pay threshold (WTP) and other socket-free interventions leading to the outline of an implementation strategy.

Supplementary material will be published in Data In Brief, including a list of scenarios, a breakdown of allowable hours for labor, components with and without the KWI for all K-levels, mapping of 36-item Short Form Survey (SF36) data into QALY, and a comparison with other socket-suspended and socket-free cost-utility studies as well as the Consolidated Health Economic Evaluation Reporting Standards (CHEERS) and the Consensus Health Economic Criteria (CHEC) extended checklists.^36–38^

## SETTING

We purposely chose to perform this preliminary CUA from the perspective of an Australian healthcare organization. Being well acquainted with BAP solutions, Australian state governments have already performed horizon scans for socket-free technologies including direct skeletal attachments that have selected the KWI.^12,19,30,35^

This choice of setting could be challenging. Indeed, Gallego et al. (2011) highlighted that the assessment of new medical devices and medical technologies prior to introduction is very difficult because of the complexity of the Australian healthcare system.^39^

However, this preliminary CUA could be facilitated by considering the same setting that we presented in Frossard et al. (2018, 2020).^19,20^ Practically, we performed this preliminary CUA from the perspective of an Australian state government Minister of Health with a yearly budget of $5 million, servicing 4,000 consumers annually through a network of up to ten prosthetists (e.g., Queensland Artificial Limb Service).^12,30,31^

## DETERMINE FEASIBILITY

As explained in Frossard (2021), this feasibility phase was organized around a three-step waterfall process with decision point at every step.^18^

### Investigate product (Step 1A)

Unlike other interventions relying on direct skeletal attachment of prosthetic limb, the KWI has no percutaneous part protruding from the skin that creates a permanent open stoma. The prosthesis is attached through a socket. Interestingly, the implant could lengthen the femoral condyle by a few centimeters depending on the bone and soft tissues conditions. The spacer restores the distal weight bearing capability on the femur, similar to a knee disarticulation.^21^ Thus, the KWI could reshape the distal end of the residuum with a more uniform and consistent cone shape that could ease socket fittings.^40–42^

All things considered, the product investigation suggested that the benefits of the KWI could be possibly translated into a reduction of socket fittings for a large population of TFAs (e.g., vascular, trauma, tumor). We found the information satisfactory to warrant further searches for evidence of safety.

### Search for evidence of safety (Step 1B)

Preliminary evidence of stability of KWI is presented in **Table 1A.**^24^ A recent cohort study (N=13) showed that the mean percentage of bone mineral density of the amputated limb in comparison with the sound limb was 70.6% pre-implantation and 73.2% 14 months post-implantation, with an average increase of 2.6%.^24^ This study suggested that femoral stem not only osseointegrates over time but also could increase cortical thickness around the implant.^43,44^

**Table 1:** Summary of clinical outcomes with and without Keep Walking Implant (KWI).

Early evidence of clinical outcomes	Without KWI (Before treatment)	With KWI (After treatment)
**A-Safety**		
** Stability**		
Percentage of bone mineral density^* 24^	70.6%	73.2%
**B-Efficacy**		
** Prosthetic use**		
Daily use	10.7 hr/day	12.9 hr/day
Houghton scale score	9.7	9.8
LCI score	38.0	39.0
** Mobility**		
Gait speed^23,25^	0.9±0.3m/s	1.1±0.3m/s
2 MWT^23,25^	103.6±34.7m	128±38.9m
PCI^25^	0.57±0.3	0.51±0.2
** Health-related quality of life**		
SF36-Physical Functioning^22^	39.0±10.1	45.8±8.1
SF36-Role Physical^22^	51.9±9.0	54.1±3.9
SF36-Bodily Pain^22^	51.1±11.8	53.3±8.1
SF36-General Health^22^	51.3±10.1	52.4±9.8

* Bone mineral density of the amputated limb expressed as a percentage with the sound limb; LCI: Locomotor Capabilities Index; 2 MWT: 2-Minute Walk Test; PCI: Physiological cost index; SF36: 36-Item Short Form Survey.

Long-term cohort studies currently being conducted will confirm to what extent this increase of stability impacts risks of loosening, periprosthetic fractures, and infections while revealing incidence of breakage of implant parts and the overall rate of implant removal. In the meantime, van Eck and McGough (2015) showed that the infection rate of osseointegrated implants relying on a percutaneous part ranged from 2% to 41%.^45,46^ In principle, the risk of infections with the KWI should be significantly lower than these osseointegrated solutions. The absence of stoma limits continuous exposure to the environment and subsequent risks of infection. Rate of infection should be comparable to hip or knee replacement procedures, that is roughly about 1%.

Regardless of evidence gaps in adverse events, we found sufficient indications that the KWI has the capacity to provide a safe prosthetic osseointegrated attachment solution to search for the evidence of efficacy.

### Search for evidence of efficacy (Step 1C)

Evidence of efficacy of KWI for cohort studies conducted during clinical trials is summarized in **Table 1B.**^22,23,25^ Preliminary studies indicated that the use of the prosthesis with the KWI increased significantly from 10.70 to 12.87 hours per day. The self-administered Houghton scale score, reflecting a person's perception of prosthetic use, also increased from 9.65 to 9.78. The self-administered Locomotor Capabilities Index score, assessing overall locomotor abilities, increased from 38.04 to 38.95. Studies showed the efficacy of the KWI to restore walking ability.^23,25^ Studies reported a significant increase in gait speed from 0.86±0.29 m/s to 1.06±0.32 m/s as well as distance walked from 103.6±34.7 m to 128±38.9 m during a two-minute walk test conducted 14 months after implantation.^23,25^ The Physiological Cost Index, representing energetic efficiency of walking, showed no significant difference without and with the KWI.^25^ Guirao et al. (2018) used the 36-item Short Form Survey (SF36) to show improvement in quality-of-life with the KWI.^22^ Participants reported that the treatment led to improvement in each health domain score, including an increase of 3.94±9.22 and 1.14±8.07 for the Summary Physical and Mental Health Components, respectively.^22^

Further evidence comparing patient's experience with prosthetic use and socket fittings using surveys like the Orthotics and Prosthetics User's Survey (OPUS), the Quebec User Evaluation of Satisfaction with assistive Technology (QUEST 2.0), and the Socket Comfort Score (SCS) without and with the KWI is needed. However, one could argue that outcomes presented above could be surrogate indicators of socket comfort. Therefore, these results suggested that KWI might contribute to increase overall socket comfort. Despite of these knowledge gaps, we believe that the outcomes showing efficacy of the KWI solution currently available were adequate to justify completing the rest of the preliminary CUA.

## OUTLINE CONSTRUCTS

This five-step phase entailed choosing the list of parameters framing this preliminary CUA.

### Define perspective (Step 2A)

A CUA from government healthcare perspective could be achieved when primary, secondary, and tertiary services of a healthcare organization are centralized and interconnected enough to produce analytics and report whole care costs.^47,48^ However, like many other systems worldwide, the structure of Australian state healthcare organizations is siloed.^39^ Each service manages its own resources often independently of other services. Whether implantation of KWI increases or reduces ongoing medical costs has little relevance to administrators of prosthetic care services.^10,19,20^ Rather, they would be more interested in knowing if the alleged capacity of the KWI to reduce socket fittings could contribute to alleviating some of the prosthetic care financial burden.^10–12^

Therefore, conducting a preliminary CUA of the KWI from the Australian government prosthetic care perspective, as were recently published studies examining prosthetic direct skeletal attachments, would be a relevant starting point.^19,20^

### Define the time horizon (Step 2B)

Some studies suggested that a rather short time horizon would be indicated for the preliminary CUA for the KWI.^14,47,49–55^ Because of the prosthetic care perspective, the time horizon should be primarily determined by realistic estimations of the costs for the fitting of the sockets as well as knee and foot units. Therefore, we chose a six-year time horizon that corresponded to a funding cycle allowing the replacement of knee and foot/ankle units at the end of their respective three and two-year expected lifespans.^19,20,56^ We assumed that estimations of components costs beyond this time horizon were likely to be grossly inaccurate. As detailed below, we considered that TFAs fitted without and with the KWI would experience steady utilities over this time horizon.

### Identify scenarios (Step 2C)

**Figure 2:**
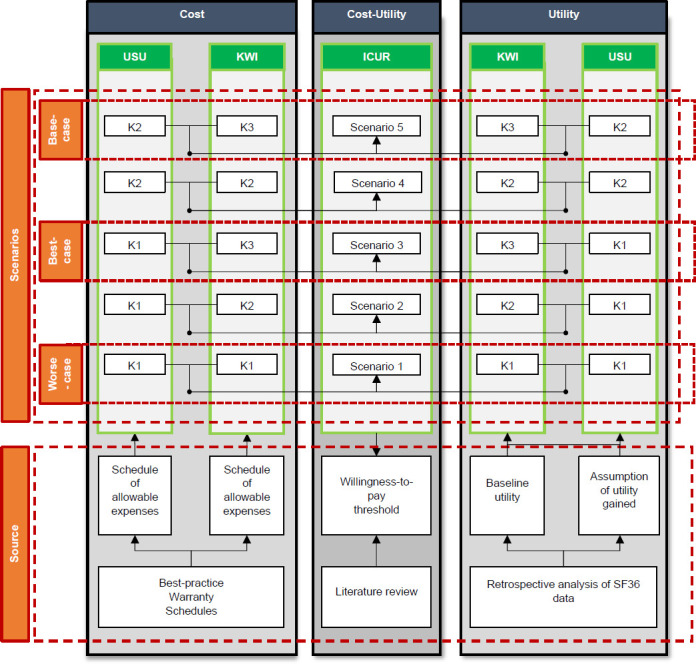
Overview of sources used to perform preliminary cost-utility analyses using incremental cost-utility ratio (ICUR) and willingness-to-pay threshold (WTP=$40,000 per QALY) to compare costs and utilities for the provision of transfemoral socket-suspended prostheses attached to residuum without (USU) and with distal weight bearing Keep Walking Implant (KWI) over a six-year time horizon for five plausible clinical scenarios considering various progressions between K-Levels (K1, K2, K3) including worse-case, base-case and best case. (SF36: 36-Item Short Form Survey).

In principle, progressions across the five Medicare functional K-Levels (K0–K4) for up to 15 scenarios could considered, as detailed in the supplementary material.^18^ However, instead, we purposely investigated only the five scenarios we deemed the most realistic and likely to represent expected clinical outcomes with the KWI as described in **Figure 2**.^22–25^ Worse-case, best-case, and base-case scenarios corresponding to scenarios 1, 3, and 5 were created assuming no progression for K1, as well as progression from K1 to K3 and K2 to K3 without and with the KWI, respectively.

### Set discount (Step 2D)

The six-year time horizon was short enough to predict costs of provision of prosthetic fittings (e.g., labor, parts). The most important costs would be incurred at the beginning of the cycle. Finally, we assumed that utilities would remain consistent across the time horizon. Consequently, no costs and utilities were discounted.

### Assess uncertainty (Step 2E)

Here, the key events were the socket fittings that we purposely reduced to one per annum with the KWI, as justified in Step 3B. We considered that comparisons of cost reduction going from four or less to one socket fitting yearly were deemed beyond the scope of this preliminary CUA. Nonetheless, impact of socket fittings frequency could be easily achieved given the readability and scalability of the raw data presented here and in the supplementary material.

Alternatively, the sensibility analysis was limited to the extraction of basic descriptive statistics (e.g., mean, standard deviation, lower and upper 95% confidence intervals, minimum, maximum, and range) for the costs, utilities, and ICURs aggregated across the scenarios.

## CONDUCT ANALYSIS

This four-step phase estimates costs, utilities, and ICURs for all selected scenarios.

### Estimate costs (Step 3A)

Primary post-treatment costs for the provision of prosthetic care with the KWI are not yet available in Australia. Alternatively, typical expenses for the provision of prosthetic care without and with the KWI were extracted from two schedules detailing allowable expenses for labor and parts (**Table 2** and **Table 3**). The type and frequency of intervention were recommended by two qualified and experienced Australian prosthetists, taking into consideration the best practices for prosthetic care with the KWI and lifetime of components. The actual dollar value of an individual item was based on recently published schedules of allowable expenses for lower limb BAP as well as prices recommended by the Australian National Disability Insurance Scheme (NDIS), as detailed in the supplementary material.^10,12,19,57^

**Table 2:** Schedule of typical allowable expenses over six-year time horizon with yearly breakdown of labor and parts costs allocated for provision of transfemoral socket-suspended prostheses attached to residuum without distal weight bearing Keep Walking Implant for each K-Level (K1, K2). The number of units for all labor-related expenses corresponded to number of hours spent by a qualified prosthetist (e.g., CPO) at the standard Australian fixed hourly rate of $180.

Tasks	Yearly cost						Total
	Y1	Y2	Y3	Y4	Y5	Y6	
**Labor and parts for socket for all K-Levels**							
Design and fit socket	$11,520	$11,520	$11,520	$11,520	$11,520	$11,520	$69,120
Fit liner	$360	$360	$360	$360	$360	$360	$2,160
Fit prosthesis	$1,800	$1,800	$1,800	$1,800	$1,800	$1,800	$10,800
Maintain prosthesis	$360	$360	$360	$360	$360	$360	$2,160
**Total labor**	**$14,040**	**$14,040**	**$14,040**	**$14,040**	**$14,040**	**$14,040**	**$84,240**
Socket valve, adapters	$1,200	$1,200	$1,200	$1,200	$1,200	$1,200	$7,200
Liner	$2,000	$2,000	$2,000	$2,000	$2,000	$2,000	$12,000
Cosmesis	$1,000	$1,000	$1,000	$1,000	$1,000	$1,000	$6,000
**Total parts**	**$4,200**	**$4,200**	**$4,200**	**$4,200**	**$4,200**	**$4,200**	**$25,200**
**Parts for K1**							
Knee	$3,000			$3,000			$6,000
Foot	$1,000		$1,000		$1,000		$3,000
Tube, clamp, pylon	$1,400			$1,400			$2,800
**Total parts for K1**	**$5,400**		**$1,000**	**$4,400**	**$1,000**		**$11,800**
**Total for K1**	**$23,640**	**$18,240**	**$19,240**	**$22,640**	**$19,240**	**$18,240**	**$121,240**
**Parts for K2**							
Knee	$13,500			$13,500			$27,000
Foot	$2,750		$2,750		$2,750		$8,250
Tube, clamp, pylon	$1,400			$1,400			$2,800
**Total parts for K2**	**$17,650**		**$2,750**	**$14,900**	**$2,750**		**$38,050**
**Total for K2**	**$35,890**	**$18,240**	**$20,990**	**$33,140**	**$20,990**	**$18,240**	**$147,490**

**Table 3:** Schedule of typical allowable expenses over six-year time horizon with yearly breakdown of labor and parts costs allocated for provision of transfemoral socket-suspended prostheses attached to residuum with distal weight bearing Keep Walking Implant for each K-Level (K1, K2, K3). The number of units for all labor-related expenses corresponded to number of hours spent by qualified prosthetist (e.g., CPO) at the standard Australian fixed hourly rate of $180.

Tasks	Yearly cost						Total
	Y1	Y2	Y3	Y4	Y5	Y6	
**Labor and parts for socket for all K-Levels**							
Design and fit socket	$5,760	$5,760	$5,760	$5,760	$5,760	$5,760	$34,560
Fit liner	$360	$360	$360	$360	$360	$360	$2,160
Fit prosthesis	$1,800	$1,800	$1,800	$1,800	$1,800	$1,800	$10,800
Maintain prosthesis	$360	$360	$360	$360	$360	$360	$2,160
**Total labor**	**$8,280**	**$8,280**	**$8,280**	**$8,280**	**$8,280**	**$8,280**	**$49,680**
Socket valve, adapters	$600	$600	$600	$600	$600	$600	$3,600
Liner	$1,000	$1,000	$1,000	$1,000	$1,000	$1,000	$6,000
Cosmesis	$1,000	$1,000	$1,000	$1,000	$1,000	$1,000	$6,000
**Total parts**	**$2,600**	**$2,600**	**$2,600**	**$2,600**	**$2,600**	**$2,600**	**$15,600**
**Parts for K1**							
Knee	$3,000			$3,000			$6,000
Foot	$1,000		$1,000		$1,000		$3,000
Tube, clamp, pylon	$1,400			$1,400			$2,800
**Total parts for K1**	**$5,400**		**$1,000**	**$4,400**	**$1,000**		**$11,800**
**Total for K1**	**$16,280**	**$10,880**	**$11,880**	**$15,280**	**$11,880**	**$10,880**	**$77,080**
**Parts for K2**							
Knee	$13,500			$13,500			$27,000
Foot	$2,750		$2,750		$2,750		$8,250
Tube, clamp, pylon	$1,400			$1,400			$2,800
**Total parts for K2**	$17,650		$2,750	$14,900	$2,750		$38,050
**Total for K2**	$28,530	$10,880	$13,630	$25,780	$13,630	$10,880	$103,330
**Parts for K3**							
Knee	$24,000			$24,000			$48,000
Foot	$4,500		$4,500		$4,500		$13,500
Tube, clamp, pylon	$1,400			$1,400			$2,800
**Total parts for K3**	**$29,900**		**$4,500**	**$25,400**	**$4,500**		**$64,300**
**Total for K3**	**$40,780**	**$10,880**	**$15,380**	**$36,280**	**$15,380**	**$10,880**	**$129,580**

In both schedules, the cost of labor corresponded to the number of hours allocated to a prosthetist for socket fittings. We considered that only a qualified prosthetist (e.g., a CPO) solely undertook all the labor at the standard Australian hourly rate of $180. Typically, NDIS recommends that a prosthetist should spend approximately 32 hours for a socket fitting including six hours to cast the residuum, 20 hours to build the socket, and six hours to fit the socket. Therefore, we allowed $5,760 for 32 hours to design and fit a socket as well as $360 for two hours to fit two liners, $1,800 for ten hours to fit the prosthesis, and $360 for two hours to maintain the prosthesis on an annual basis. The cost of a part corresponded to the typical portion of the total cost that is more likely to be subsidized by the government. We allowed $600 for parts per socket (e.g., socket valve, adapters), $1,000 for liners or sleeves, $1,400 for parts per prosthesis (e.g., tube, clamp, pylon) and $1,000 for basic cosmesis each year.

As recommended by the NDIS, we allowed $3,000, $13,500, and $24,000 toward the provision of a knee unit every three years as well as $1,000, $2,750, and $4,500 toward the provision of a foot/ankle unit every two years for K1, K2, and K3 cases, respectively. Government organizations such as NDIS support the provision of categories of liners, sleeves, knees (e.g., single axis cadence responsive knee, affordable microprocessor-controlled knees), and feet/ankles (e.g., dynamic foot, energy storing and return) depending on functional levels.^32,58^ Prescription of components is left to the prosthetist, who chooses a model and brand accordingly to the patient's specific needs. Thus, we purposely allocated lump sums rather than price tags for specific prosthetic components.

Schedules differed by the number of sockets allowed per year. We assumed that K1 and K2 cases experienced issues with their residuum and sockets fittings before the intervention that were significant enough to require two socket fittings per year (**Table 2**). We also hypothesized that the clinical benefits of the KWI should translate into a reduction of socket fittings from two to one per year for K1, K2, and K3 cases (**Table 3**). We conservatively reduced and limited the yearly frequency of socket fittings down to one with the KWI to match the minimal provision supported by some healthcare systems.

The total cost across all scenarios was $131,740±$14,378 without KWI and $108,580±$21,962 with the KWI, giving an incremental cost reduced by $23,160±$21,962 across all scenarios over the six-year time horizon (**Table 4**).

**Table 4:** Overview of total costs, utilities, incremental cost-utility ratio (ICUR) and differences between ICUR and willingness-to-pay threshold (WTP=$40,000 per QALY) for yearly provision of two and one sockets fitted transfemoral prostheses attached to residuum without (USU) and with distal weight bearing Keep Walking Implant (KWI) for each K-Level (K1, K2, K3) over a six-year time horizon, respectively. (Scenarios 1: Worse-case, Scenarios 3: Best-case, Scenarios 5: Base-case).

Scenario	USU			KWI			Incremental cost	Incremental utility	ICER	Below WTP
	K-Level	Cost	Utility	K-Level	Cost	Utility				
		($)	(QALY)		($)	(QALY)	($)	(QALY)	($/QALY)	($/QALY)
Scenario 1	K1	$121,240	4.443	K1	$77,080	4.868	−$44,160	0.424	−$104,033	−$144,033
Scenario 2	K1	$121,240	4.443	K2	$103,330	4.969	−$17,910	0.526	−$34,056	−$74,056
Scenario 3	K1	$121,240	4.443	K3	$129,580	5.071	$8,340	0.627	$13,295	−$26,705
Scenario 4	K2	$147,490	4.585	K2	$103,330	4.969	−$44,160	0.384	−$114,975	−$154,975
Scenario 5	K2	$147,490	4.585	K3	$129,580	5.071	−$17,910	0.485	−$36,890	−$76,890
**Mean**		**$131,740**	**4.500**		**$108,580**	**4.990**	**-$23,160**	**0.489**	**-$55,332**	**-$95,332**
**SD**		**$14,378**	**0.078**		**$21,962**	**0.085**	**$21,962**	**0.094**	**$53,459**	**$53,459**
Lower 95%CI		$119,138	4.432		$89,330	4.915	−$42,410	0.407	−$102,190	−$142,190
Upper 95%CI		$144,342	4.568		$127,830	5.064	−$3,910	0.572	−$8,474	−$48,474
Min		$121,240	4.443		$77,080	4.868	−$44,160	0.384	−$114,975	−$154,975
Max		$147,490	4.585		$129,580	5.071	$8,340	0.627	$13,295	−$26,705
Range		$26,250	0.142		$52,500	0.203	$52,500	0.243	$128,270	$128,270

### Estimate utilities (Step 3B)

Actual utility information is also yet to be available for a cohort of Australians. We overcame this lack of primary utilities by analyzing outcomes of SF36 provided by Guirao et al (2018) identified during Step 1C.^22^ The authors recorded the utilities four months pre-operatively without the KPI and 14 months post-surgery with the KPI. A total of 23 individuals with unilateral TFA fitted with the KWI between March 2011 and November 2014 participated to this multicenter clinical study in Spain (females: five (22%), males: 18 (78%); age: 52.65±15.6 years; height: 1,66±0,93 m; mass: 67.97±11.96 kg; BMI: 24.51±2.74 kg/m2; trauma: 11 (48%), oncologic: three (13%), vascular: nine (40%); clinical trial registration: 358/10/EC). Key inclusion criteria for the recruitment were prior fitting of prosthesis for at least 12 months, prosthetic use for more than six hours per day, ability to walk indoors with or without supervision and ambulation aids, and unsatisfactory use of socket.

The mapping of the SF36 data into QALY required information that was only partially presented in the initial publication. However, the authors provided all the raw data required to complete the analysis. First, the raw SF36 data were processed to produce the mean scores for the eight health dimensions as well as the physical and mental components summary scores. Next, each SF36 dataset without and with the KWI was converted into QALY applying the Ara and Brazier regression model also used by Frossard et al (2018).^19,59^ This provided a baseline utility of 0.788 and 0.845 QALY per year for provision of prosthetic care without and with the KWI, respectively.

K-Level classification during the recording of SF36 data was beyond the scope of the initial study.^22^ However, we prudently assumed that these baseline utilities were most likely to be experienced by K3 cases without and with the KWI, based on the aforementioned recruitment criteria (e.g., prosthetic use for more than six hours per day, ability to walk indoors with or without supervision, and ambulation aids).

Finally, we cautiously assumed that K1 and K2 cases without KWI as well as K1 and K2 cases with the KWI might experience 6% and 3% as well as 4% and 2% less utilities than baselines, respectively (**Table 5**). Finally, the estimated utility values were cumulated over the six-year time horizon to provide total gain of QALYs. We considered that the estimated utility values would remain unchanged over the time horizon like the health-related quality of life data reported by Hagberg for a cohort of individuals with transfemoral BAP over a 15-year follow-up study.60

**Table 5:** Assumed percentage of utility gained in relation of baseline of 0.788 QALY per year and 0.845 QALY per year extracted from Guirao et al (2018) as well as total QALY for time horizon following the provision of transfemoral socket-suspended prostheses attached to residuum without (USU) and with distal weight bearing Keep Walking Implant (KWI) for each K-Level (K1, K2, K3), respectively.^22^

	Percentage of decrease	QALY per year	QALY for time horizon
**USU**			
K1	−6	0.741	4.443
K2	−3	0.764	4.585
K3	0	0.788	4.727
**KWI**			
K1	−4	0.811	4.868
K2	−2	0.828	4.969
K3	0	0.845	5.071

The mean cumulated utility across all scenarios was 4.500±0.078 QALY without KWI and 4.990±0.085 QALY with the KWI giving an incremental utility increased by 0.489±0.094 QALY over the six-year time horizon (**Table 4**).

### Calculate incremental cost-utility ratios (Step 3C)

**Figure 3:**
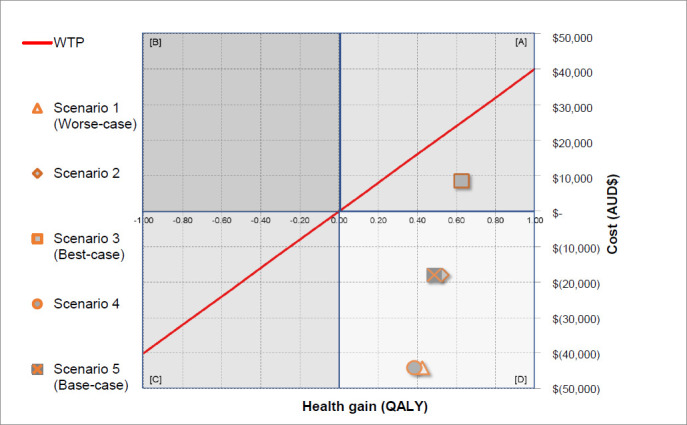
Cost-utility analysis showing incremental cost-utility ratio (ICUR) including the indicative ICUR of −$36,890 per quality-adjusted life-year (QALY) for the best-case and willingness-to-pay threshold (WTP) of $40,000 per QALY for the provision transfemoral socket-suspended prostheses attached to residuum without and with distal weight bearing Keep Walking Implant (KWI) including quadrants indicating that provision with the KWI was more costly and more effective (Quadrant A: Consider ICUR), more costly and less effective (Quadrant B: Dominated), less costly and less effective (Quadrant C: Consider ICUR), less costly and more effective (Quadrant D: Dominant) than usual intervention.

ICURs was calculated using the formula ICUR = (Cost with the KWI – Cost without KWI)/(Utility with the KWI – Utility without KWI).^18^ Individual ICUR was calculated for each scenario. We considered that an indicative ICUR corresponded to a base-case scenario. All ICURs were plotted on a conventional cost-effectiveness plane diagram (**Figure 3**).^17^

The mean ICUR across all scenarios was −$55,332± $53,459 per QALY (**Table 4, Figure 3**). The provision of prosthetic care with the KWI was more costly and more effective than usual intervention only for base-case with an ICUR of $13,295 per QALY. Prosthetic care with the KWI was less costly and more effective than usual intervention for all the other scenarios including worse-case and base-case with a mean ICUR of −$72,489±$42,990 per QALY. The indicative ICUR corresponding to base-case was-$36,890 per QALY.

### Compare with willingness-to-pay threshold (Step 3C)

The oft-cited WTP is $50,000 per QALY.^17^ We applied a conservative threshold of $40,000 per QALY, as suggested by the Australian Pharmaceutical Benefits Advisory Committee.^19^

The mean ICUR across all scenarios was $95,332±$53,459 per QALY below WTP (**Table 4, Figure 3**). The ICUR for the provision of prosthetic care with the KWI corresponding to base-case was $26,705 per QALY below WTP. The ICUR for prosthetic care with the KWI for all the other scenarios including worse-case and base-case was $112,489± $42,990 per QALY below WTP. The indicative ICUR was $76,890 per QALY below WTP.

## INTERPRET OUTCOMES

This three-step phase established whether the outcomes of this preliminary CUA should facilitate or curtail further clinical introduction of the KWI in Australia.

### Consider limitations (Step 4A)

This study presented the typical limitations of preliminary CUAs mentioned in Frossard (2021) (e.g., narrow perspective, simple scenarios, short time horizon, strong assumptions, data mismatch, lack of uncertainty data).^13,18^

However, our assumptions resulted from educated choices made erring on the side of caution to estimate costs and utilities for a series of plausible scenarios. We prudently considered that the KWI could reduce socket fittings by only one compared to the usual intervention.

Consequently, some costs for labor and parts might have been overestimated. Only a qualified prosthetist performed socket fittings, although some tasks could be undertaken by a technician paid at a lower hourly rate. We assumed that the full allowable expenses would be claimed, although previous studies demonstrated that consumers could spend less by choosing to keep using components after the warranty expired and/or overlook services and components (e.g., cosmetic covers).^19,32^ No costs were discounted.

Furthermore, utilities might have been underestimated. We conservatively credited baseline utility to K3 cases, allocated low incremental gains, and considered utilities gained post-treatment consistent over the years. However, the baseline utility was calculated retrospectively for a population in a jurisdiction where the perception of living without a limb, and therefore QALY, might be different than in Australia. This cohort study had a small but reasonable sample size (N=23) representing approximately 30% of the existing population fitted with the KWI.^22–25,61,62^

Finally, the interpretation of the ICURs was limited by aggregation of costs and utilities with mismatched sources (i.e., estimated vs. real), jurisdictions (i.e., Australia vs. Spain), onset (i.e., 2018–2019 price vs 2011–2014 recruitment) and post-operative timeline (i.e., six-years vs. 14 months), respectively. However, we considered a conservative WTP that was approximately 20% or $10,000 below typical WTP.

### Interpret outcomes (Step 4B)

The outcomes produced with the series of assumptions ascertained that the reduction of costs by 0.83±0.17 folds or $23,160±$21,962 combined with reasonable increase of 1.11±0.02 folds or 0.489±0.094 QALY could make the implantation of the KWI cost-effective and noticeably below WTP over a six-year time horizon from an Australian governmental prosthetic care perspective.

Outcomes could be compared to recent CUAs focusing on socket-suspended and socket-free BAP solutions that were also performed with the same constructs, as detailed in the supplementary material.^19,20,57^

The costs for labor and parts, including knees and feet, were comparable to those considered in Frossard et al (2017, 2018, 2020).^12,19,20^ However, baseline utility without and with the KWI extracted from Guirao et al (2018) was 0.145 QALY or 0.225 folds and 0.202 QALY or 0.314 folds higher than the utilities experienced with socket-suspended and BAP before and after implantation of osseointegrated percutaneous device reported in Frossard et al (2018), respectively.^19,22^ The incremental utility extracted from Guirao et al (2018) was 0.586 QALY or 0.911 folds less than the one reported in Frossard et al (2018).^19,22^ Interestingly, the average incremental utility across all the scenarios we considered in this study was 0.562±0.016 QALY or 0.873±0.024 folds less than the incremental QALY presented in Frossard et al (2018).^19^ These comparisons confirmed that our estimations of costs and utilities for the provision of prosthetic care with the KWI were sensible.

The proposed indicative ICUR for the provision of prosthetic care without and with the KWI was $125,099 per QALY or

5.94 folds, $445 per QALY or 0.02 folds, and $53,522 per QALY or 2.54 folds less costly than the ICUR for the provision of BAP in the worse-case, best-case, and case-base presented in Frossard et al (2018), respectively.^19^ The differences between the base-cases might be due to clinical guidelines recommending that BAP should be fitted with advanced and costlier microprocessor-controlled knees and energy storing and return feet to protect the fixation (e.g., increase stance phase stability, avoid excessive loading, prevent falls) and reduce adverse events (e.g., periprosthetic factures, mechanical failures).^32,58,63–66^

These comparisons suggested that the KWI has the potential to be more cost effective than current socket-suspended and other osseointegrated solutions, particularly when considering the base-case and worse-case scenarios. However, generalization of the outcomes intrinsic to the KWI must be considered carefully. The post-treatment baseline utility might be used in other studies. The costs extracted from Australian-specific schedules might only be partially transferable to other jurisdictions worldwide, particularly in European and North American countries.^7,52,54,67–70^

### Outline implementation strategy (Step 4C)

The indicative ICUR appeared to stack up favorably against other socket-suspended or socket-free solutions currently available from an Australian government prosthetic care perspective. Furthermore, all ICURs presented here were below the $20,000 per QALY threshold, making an innovation most likely to be recommended for clinical introduction, as described in Frossard (2021).^18^

In sum, this preliminary CUA provided sufficient favorable evidence to justify recommending market access and clinical introduction of the KWI, at least from an Australian healthcare perspective. However, interpretation of these outcomes could be easily transferable to other healthcare organizations with a similar ethos worldwide.

Identifying pathways for the clinical introduction was beyond the scope of this study (e.g., training clinicians, testing site, registration of clinical trials, selection of participants). However, this study could inform subsequent full CUAs of the KWI to be conducted within-trial horizon and beyond the trial follow-up for patients.^49^

Primary CUAs could consider true costs extracted from financial systems and utilities measured regularly with standard surveys for cohorts of participants treated without and with the KWI. In principle, prospective primary study could take several years because of the usual time required to implement the surgical procedure (e.g., clinical trial registration, ethical approval, recruitment of participants, surgical procedures, learning curve of practitioners, observation time between procedures). Alternatively, primary analyses could aggregate actual and generic costs presented here and in other recent studies.^19,20^

Meanwhile, modeling CUAs could consider costs and utilities presented here to modify or develop specific Bayesian or Markov models.^51^ These analyses could predict the outcomes of KWI from broader health care perspectives aggregating utilities as well as fixed and ongoing surgical, medical, and prosthetic care costs over a scalable timeline (e.g., lifetime), assuming that issues with siloed healthcare financial systems could be overcome.

Assessments of health economic benefits of KWI using either primary or modeling approaches will benefit from stratified analyses considering a wide range of case-mixes with various demographics (e.g., young vs. elderly), causes of amputation (e.g., vascular vs. non-vascular), functional levels (e.g., K-Level), attachments (e.g., socket vs. BAP), multiple prosthetic fittings with liners, knees and ankles/feet (e.g., basic vs. advanced components) from healthcare, and societal perspectives (e.g., return to work).^34,47,70,71^

## CONCLUSIONS

A preliminary CUA comparing the provision of prosthetic care, particularly socket fittings, without (usual intervention) and with the KWI (new intervention) was performed for the first time. Practically, this preliminary CUA provided administrators of healthcare organizations in Australia and elsewhere worldwide with prerequisite evidence justifying further market access and clinical promotion of the KWI. More broadly, this work indicates that a basic framework of preliminary CUA of prosthetic care innovation proposed previously is not only feasible but also informative when a series of assumptions is carefully considered. This study further confirms that preliminary CUAs might be a relevant alternative to full CUA prosthetic care interventions, like any other medical treatment.

## CALL TO ACTION

- Share these outcomes with healthcare administrators Australia and similar healthcare organization worldwide responsible for facilitating access to market of KWI solution.
- Suggest authors of health economic evaluations to use the information provided in this preliminary CUAs and others to benchmark new innovations susceptible to ease prosthetic and, more particularly, socket fittings.
- Stimulate discussion amongst authors of health economic evaluations on how to capitalize on the lessons learnt from recent experiences including this work to identify a series of manageable barriers and transferrable facilitators of preliminary CUAs of prosthetic care innovations.

## DECLARATION OF CONFLICTING INTERESTS

Lluis Guirao is currently Chief of the Rehabilitation Service - Hospital Asepeyo Sant Cugat, Barcelona, Spain. Lluis Guirao is one of the inventors of the Keep Walking Implant. He receives no monetary compensation related to the Keep Walking Implant. Beatriz Samitier is currently Rehabilitation Specialist at Servicio de Rehabilitaión - Hospital Asepeyo Sant Cugat, Barcelona, Spain. Laurent Frossard, Director and Chief Scientist Officer of YourResearchProject Pty Ltd, was appointed as consultant by TEQUIR S.L. to manage this project of research including collection, analysis, and reporting cost-effectiveness data. He has worked as consultant for several organizations on non-related educational programs and projects of research focusing on recording loading data, developing of database to record clinical outcomes as well as drafting grants and manuscripts for Cognitive Institute, Exercise & Sports Science Australia, Griffith University, iPug Pty Ltd, Middlesex University, New Zealand Artificial Limb Service, Osseointegration Group of Australia Pty Ltd, OSSUR, Poly-Orthodox International, Queensland Artificial Limb Service, Queensland University of Technology, Return to Work-South Australia, South Australia Health, TEQUIR S.L, University of the New South Whales, University of the Sunshine Coast.

## SOURCES OF SUPPORT

This study was partially funded by TEQUIR S.L. and Stable Orthopaedics Pty Ltd. These companies had no influence on the design, data analysis, or interpretation of this research study. TEQUIR S.L. provided technical information about the Keep Walking Implant as well as the raw quality of life data and contributed to the writing of this manuscript.

## LIST OF ABBREVIATIONS

BAP: Bone-anchored prostheses
BMI: Body mass index
CUA: Cost-utility analysis
CER: Incremental cost-effectiveness ratio
ICUR: Incremental cost-utility ratio
K1: Individuals classified in Level 1 of Medicare Functional Classification
K2: Individuals classified in Level 2 of Medicare Functional Classification
K3: Individuals classified in Level 3 of Medicare Functional Classification
K4: Individuals classified in Level 4 of Medicare Functional Classification
K-Level: Medicare Functional Classification Level
K0: Individuals classified in Level 0 of Medicare Functional Classification
KWI: Distal weight bearing Keep Walking Implant
L-Code: Procedure extracted from US Healthcare Common Procedure Coding System
QALY: Quality-adjusted life-year
SF36: 36-Item Short Form Survey
WTP: Willingness-to-pay threshold
